# Diagnostic Value of Inflammatory Biomarkers in Differentiating Vascular Dementia From Alzheimer's Disease: A Systematic Review and Meta‐Analysis

**DOI:** 10.1002/brb3.71341

**Published:** 2026-04-16

**Authors:** Yingchun Ling, Jiao Sun, Lingmin Hu, Mingyong Zhao, Jie Chen, Yating Zheng

**Affiliations:** ^1^ Department of Clinical Laboratory Shaoxing Seventh People's Hospital Shaoxing City China; ^2^ Department of Pharmacy Shaoxing Seventh People's Hospital Shaoxing City China; ^3^ Department of Geriatrics Shaoxing Seventh People's Hospital Shaoxing City China

**Keywords:** Alzheimer's disease, inflammatory biomarkers, meta‐analysis, systematic review, vascular dementia

## Abstract

**Introduction:**

Dementia encompasses distinct subtypes characterized by different underlying mechanisms—most notably, Alzheimer's disease (AD), driven by neurodegeneration, and vascular dementia (VaD), stemming from cerebrovascular pathology. Growing evidence emphasizes the critical role of neuroinflammatory processes in both conditions, highlighting inflammatory biomarkers as potential tools for differential diagnosis. This study assesses the diagnostic accuracy of inflammatory biomarkers in distinguishing AD from VaD.

**Method:**

A systematic search of PubMed, EMBASE, Web of Science, and Cochrane Library databases was conducted in November 2025 to identify eligible studies. Standardized mean differences (SMDs) with 95% confidence intervals (CIs) were calculated to compare inflammatory marker levels between AD and VaD groups. Random‐effects models were applied for all meta‐analyses.

**Results:**

Fifteen observational studies involving 1728 participants were included. Pooled analyses showed no significant differences in IL‐6 (SMD: −0.13; 95% CI: −0.44 to 0.19; *p* = 0.433), TNF‐α (SMD: −0.22; 95% CI: −0.71 to 0.28; *p* = 0.388), or CRP (SMD: 0.73; 95% CI: −0.17 to 1.62; *p* = 0.111) between AD and VaD overall. However, subgroup analyses indicated context‐dependent variations: IL‐6 and TNF‐α levels were lower in patients with AD in studies conducted in Eastern regions, with larger sample sizes (≥100 participants), older populations (≥70 years), or higher methodological quality. CRP showed similar patterns in larger or higher‐quality studies. Importantly, IL‐1β levels were significantly higher in patients with AD compared to patients with VaD (SMD: 0.48; 95% CI: 0.18 to 0.79; *p* = 0.002).

**Conclusions:**

Exploratory analyses suggest IL‐1β as a promising candidate for differentiating AD from VaD, warranting validation in larger, prospective studies. The preliminary, context‐dependent signals for IL‐1β, IL‐6, and TNF‐α indicate that inflammatory pathways differ between these dementias but are substantially influenced by methodological and population factors.

**Systematic review registration:**

INPLASY platform (number: INPLASY202570068)

## Graphical abstract

This systematic review and meta‐analysis evaluated the diagnostic potential of inflammatory biomarkers for differentiating Alzheimer's disease (AD) from vascular dementia (VaD). Based on 15 observational studies involving 1728 participants, pooled analysis showed no significant overall differences in IL‐6, TNF‐α, or CRP levels between AD and VaD. However, subgroup analyses revealed context‐dependent patterns: IL‐6 and TNF‐α were lower in AD in studies conducted in Eastern regions, with larger sample sizes (≥100), older populations (≥70 years), or higher methodological quality, and similar trends were observed for CRP. Notably, IL‐1β levels were significantly higher in AD compared to VaD, suggesting its association with AD‐specific neuroinflammatory pathways, potentially related to amyloid‐driven inflammasome activation. These findings position IL‐1β as a promising candidate biomarker for differential diagnosis, while highlighting the substantial influence of study and population characteristics on inflammatory profiles. Further validation through large‐scale, prospective studies using standardized protocols is warranted to determine the clinical utility of inflammatory markers in distinguishing dementia subtypes.

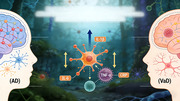

## 1 Introduction

With global population aging accelerating, the World Health Organization (WHO) projects that by 2050, 2.1 billion individuals will be aged 60 or older. Dementia has become a major public health concern, profoundly affecting the well‐being of older adults. The number of people living with dementia is expected to rise from 55 million in 2019 to approximately 139 million by 2050 (World Health Organization [WHO] 2025). Alzheimer's disease (AD) and vascular dementia (VaD) together account for 60%–80% of dementia cases (Soria Lopez et al. 2019; Rundek et al. 2022), yet they are defined by fundamentally different pathological mechanisms and clinical profiles (Tomimoto et al. 2004). AD is marked by β‐amyloid (Aβ) plaque accumulation and neurofibrillary tangles composed of hyperphosphorylated tau protein, while VaD results from cerebrovascular disease leading to cerebral perfusion deficits and ischemic brain injury (Lyu et al. 2024). Research indicates that targeted, subtype‐specific interventions can delay functional decline by 18–24 months, underscoring the clinical importance of accurate differential diagnosis (Morovic et al. 2019).

Current diagnostic approaches—relying on cognitive tests, structural neuroimaging, and neuropsychological evaluations—face notable limitations, particularly in the early stages of dementia (Vollhardt et al. 2025). Recent longitudinal evidence illustrates these challenges. A 2023 multicenter cohort study of 1243 prodromal patients reported that conventional diagnostic methods achieved only 65%–78% accuracy in early detection, with misdiagnosis rates as high as 22% due to overlapping clinical features between AD and VaD (Jørgensen et al. 2020). Even PET imaging, considered a diagnostic gold standard, failed to differentiate subcortical VaD from early AD in 31% of cases with mixed vascular and neurodegenerative pathology (Minoshima et al. 2021). Such diagnostic uncertainty directly impacts treatment outcomes. A 2022 retrospective analysis of 897 patients with dementia revealed that 34% received inappropriate therapies due to subtype misclassification. Specifically, patients with VaD incorrectly prescribed Aβ‐targeted treatments experienced a 2.3‐fold increase in adverse events (Van De Glind et al. 2013).

These diagnostic challenges underscore the critical need for objective, pathophysiologically grounded biomarkers. In this pursuit, research has expanded beyond classical circulating proteins to include extracellular vesicles (EVs)—particularly exosomes derived from neurons and glia. EVs can cross the blood–brain barrier and carry CNS‐specific molecular cargo, offering a more direct reflection of brain pathology than peripheral cytokines alone (András et al. 2017). Recent meta‐analyses have begun to consolidate evidence on EV‐associated biomarkers, such as phosphorylated tau in AD, showing promising diagnostic specificity (Zheng et al. 2025). However, the comparative utility of EVs or their inflammatory cargo in distinguishing AD from VaD remains a nascent and vital area of investigation.

Emerging research points to inflammatory pathways as central to the development and progression of both AD and VaD (Shan et al. 2025; Gulmammadli et al. 2022). Inflammatory biomarkers, which objectively reflect systemic inflammation, offer non‐invasive, dynamic monitoring potential and represent promising diagnostic tools for distinguishing between AD and VaD. Notably, elevated plasma interleukin‐6 (IL‐6) and tumor necrosis factor‐α (TNF‐α) levels have been linked to AD progression, while VaD exhibits distinct chemokine signatures, such as upregulated CCL2 and CX3CL1 (Zhang et al. 2025). However, study results remain inconsistent, particularly regarding IL‐6, due to methodological variability and biomarker fluctuations across disease stages.

To address this gap, we conducted a systematic review and meta‐analysis to synthesize available evidence on the diagnostic value of inflammatory biomarkers in differentiating AD from VaD. Our aim is to provide a scientific basis for integrating biomarker assessment into early, precision‐driven dementia diagnostics in clinical practice.

## 2 Methodology

### 2.1 Search Strategy and Selection Criteria

This study followed the Preferred Reporting Items for Systematic Reviews and Meta‐Analyses (PRISMA) guidelines to ensure methodological rigor and reliability of findings (Page et al. 2021). Our study was registered in INPLASY platform (number: INPLASY202570068). It focused on comparing inflammatory biomarker levels between AD and VaD. To capture all relevant evidence, no language restrictions were applied during study selection.

A comprehensive and systematic search was conducted across four major databases—PubMed, Embase, Web of Science, and the Cochrane Library—from inception up to November 2025. The search strategy was designed to be maximally inclusive. It combined controlled vocabulary and free‐text keywords across three core conceptual blocks: (1) Disease Block: Terms for AD (e.g., “Alzheimer Disease”[MeSH], AD, “Alzheimer*”); (2) Dementia Subtype Block: Terms for vascular cognitive disorders, including “Vascular Dementia”[MeSH], VaD, “Vascular Cognitive Impairment and Dementia” (VCID), “vascular cognitive disorder*”, “multi‐infarct dementia”, and “subcortical ischemic vascular dementia”; and (3) Biomarker Block: A broad set of terms covering inflammation and specific biomarkers. This included the general term “Inflammation” [MeSH] as well as an extensive list of individual inflammatory cytokines, chemokines, and acute‐phase reactants. Specifically, the search encompassed, but was not limited to: “C‐Reactive Protein”, CRP, “Interleukin‐1”, IL‐1β, “Interleukin‐6”, IL‐6, “Tumor Necrosis Factor‐alpha”, TNF‐α, “Interleukin‐8”, IL‐8, “Interferon‐gamma”, IFN‐γ, “Interleukin‐17”, IL‐17, “Interleukin‐12”, IL‐12, “Interleukin‐10”, IL‐10, “Chemokine CCL2”, MCP‐1, and others. These blocks were combined using the Boolean operator “AND”. The full, unabridged search syntax for each database, demonstrating the application of these principles, is provided in Supporting Information 1. Reference lists of relevant reviews and meta‐analyses were also manually screened to ensure comprehensive coverage.

Study selection was performed independently by two reviewers. Discrepancies were resolved through discussion, source checking, or consultation with a third senior reviewer. Studies were included based on the following criteria: (1) Population: clinically diagnosed patients with AD and patients with VaD; (2) Exposure: Reported quantitative data for at least one inflammatory biomarker (e.g., CRP, TNF‐α, IL‐6). Based on their broad relevance in dementia inflammation literature and the frequency of reporting in preliminary searches, IL‐6 was pre‐specified as the primary biomarkers, while TNF‐α, CRP, and IL‐1β were pre‐specified as secondary biomarkers; and (3) Study design: Observational studies (cross‐sectional, case‐control, or cohort) to ensure evidence homogeneity.

### 2.2 Data Collection and Quality Assessment

After study selection, data extraction was independently performed by two researchers following a standardized protocol. Both had prior systematic training in dementia and inflammation research, including 12 h of structured instruction covering: (1) Standardized variable interpretation; (2) application of the data extraction template through case exercises; and (3) calibration exercises to resolve ambiguities and establish inter‐rater reliability.

Extracted data included: Primary author's surname, publication year, study design (cross‐sectional, case‐control, or cohort), geographical region, sample size, participant age (range or mean), proportion of male participants, biomarkers analyzed, biofluid type, diagnostic criteria for AD and VaD, and key effect estimates with 95% confidence intervals (CIs). For missing data, corresponding authors were contacted. Inconsistent or unclear data were verified against original source documents and methodological appendices, with all resolutions documented in an audit trail.

To ensure methodological quality, both researchers conducted a pre‐assessment on five representative studies to calibrate Newcastle–Ottawa Scale (NOS) scoring, achieving a satisfactory intra‐class correlation coefficient before full evaluation. The NOS assessed three domains: (1) Selection (4 criteria), evaluating population representativeness and selection appropriateness; (2) Comparability (1 criterion), assessing control of confounding factors; and (3) Outcome/Exposure (3 criteria), verifying outcome assessment validity. Total NOS scores ranged from 0 to 9, with higher scores indicating better methodological quality (Wells et al. 2009). Any disagreements in data extraction or quality assessment were resolved through consensus discussions with a third senior reviewer, ensuring data accuracy and consistency before meta‐analysis.

### 2.3 Statistical Analysis

Associations between inflammatory biomarkers and AD/VaD were evaluated using standardized mean differences (SMDs) with 95% CIs. To address heterogeneity related to sample characteristics, measurement methods, and geographic differences, all meta‐analyses employed random‐effects models. These models integrate effect sizes through weighted averages while accounting for between‐study variability, enhancing the generalizability and robustness of results (DerSimonian and Laird 1986; Ades et al. 2005).

Heterogeneity was assessed using the *I* ^2^ statistic and Cochran's *Q*‐test. Significant heterogeneity was defined as *I* ^2^ ≥50% or a *Q*‐test *p*‐value <0.10. The *I* ^2^ statistic quantifies the proportion of total variation attributable to heterogeneity, while the *Q*‐test assesses the statistical significance of between‐study differences (Deeks et al. 2008; Higgins et al. 2003). Sensitivity analyses were performed by sequentially excluding individual studies and recalculating pooled estimates to evaluate result stability and explore sources of heterogeneity (Tobias 1999). Subgroup analyses were conducted based on region, sample size, mean age, biofluid type, and study quality. Differences between subgroups were assessed using interaction tests (Altman and Bland 2003). All subgroup analyses and sensitivity analyses are explicitly defined as exploratory, hypothesis‐generating analyses. Their purpose is to investigate sources of heterogeneity and identify potential moderators, not to draw definitive conclusions.

Publication bias was evaluated through visual inspection of funnel plot symmetry and formal testing using Egger's and Begg's methods (Egger et al. 1997; Begg and Mazumdar 1994). All reported *p*‐values for pooled effect estimates were two‐sided, with a significance threshold set at 0.05. Analyses were performed using STATA 12.0 (StataCorp LP, College Station, TX, USA), ensuring reproducibility and statistical rigor.

## 3 Results

### 3.1 Literature Search

**FIGURE 1:**
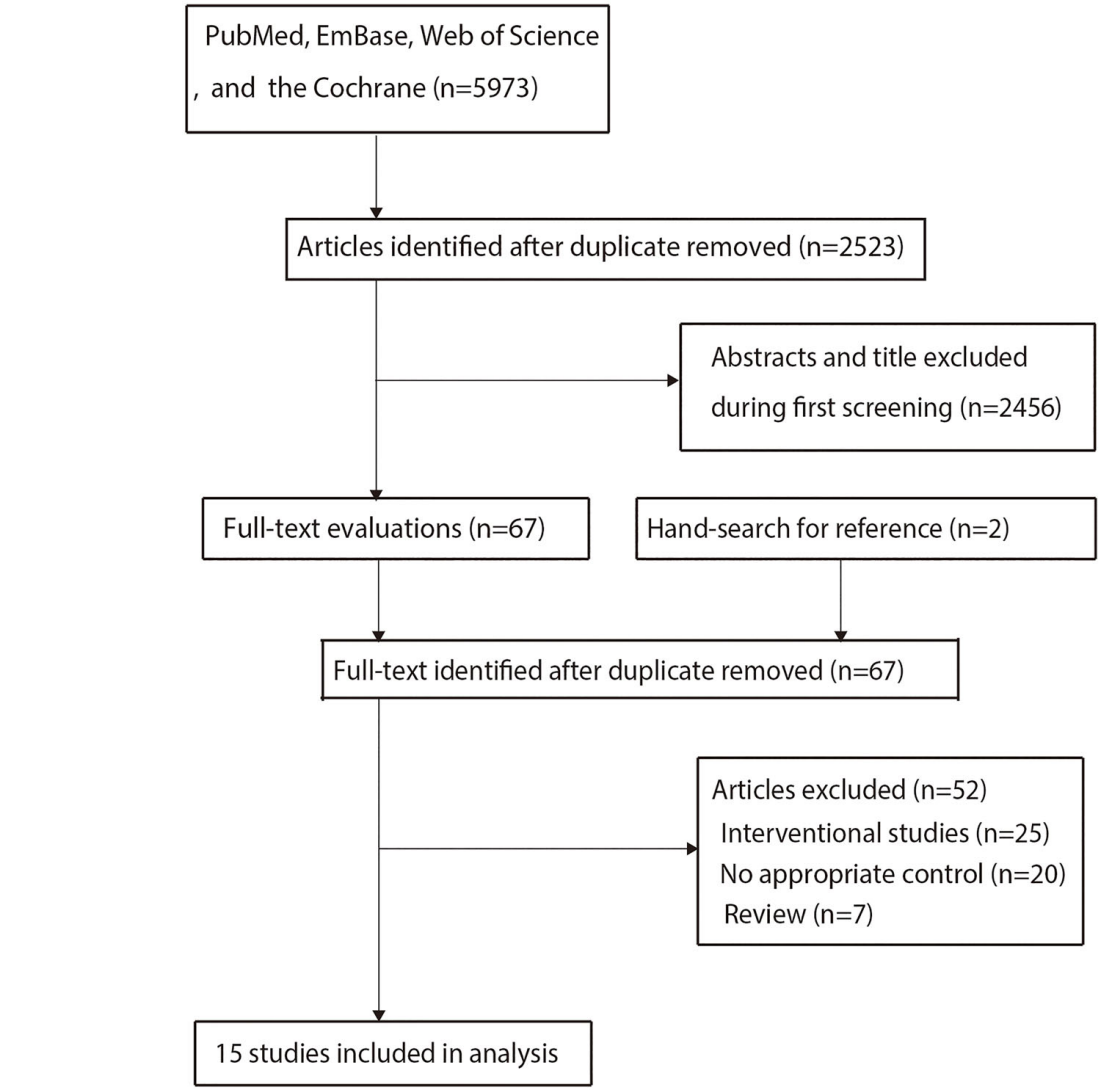
PRISMA flowchart illustrating the literature search and study selection process.

The study selection process is summarized in Figure 1. An initial search identified 5742 articles. After removing duplicates, 2523 records remained. Following title and abstract screening, 2456 studies were excluded as irrelevant. Full‐text review of the remaining 67 articles led to the exclusion of 52 more, leaving 15 studies for final inclusion in the meta‐analysis (Tarkowski et al. 1999; De Luigi et al. 2001; Paganelli et al. 2002; Wada‐Isoe et al. 2004; Jia et al. 2005; Zuliani et al. 2007; Mancinella et al. 2009; L. Li et al. 2010; Uslu et al. 2012; Dukic et al. 2016; Vishnu et al. 2017; Wehr et al. 2019; Chua et al. 2020; Lauriola et al. 2021; Khatri et al. 2024). Manual screening of reference lists from these studies identified no additional eligible studies. The general characteristics of the included studies are presented in Table 1.

**TABLE 1:** Baseline characteristics of identified studies and involved participants.

Study	Study design	Country	Sample size (AD/VaD)	Mean age (years)	Male (%)	Inflammatory biomarkers	Diagnostic criteria for AD and VaD	Study quality
Tarkowski et al. (1999)	Cross‐sectional	Sweden	67 (34/33)	67.4	55.2	TNF‐α, IL‐1β, IL‐6 (serum, ELISA)	AD: dementia with predominant instrumental deficits, as well as loss of memory, but without clinical evidence of frontal lobe dementia, dementia of the Lewy body type, or cerebrovascular changes such as white matter lesions; VaD: NINDS‐AIREN criteria	6
De Luigi et al. (2001)	Cross‐sectional	Switzerland	51 (44/7)	NA	NA	TNF‐α, IL‐1β, IL‐1Ra, IL‐6 (plasma, ELISA)	AD: MINCDS‐ADRDA criteria; VaD: modified HIS>4	5
Paganelli et al. (2002)	Cross‐sectional	Italy	54 (36/18)	76.0	35.2	TNF‐α, IL‐1β (serum, ELISA)	AD: MINCDS‐ADRDA criteria; VaD: NINDS‐AIREN criteria	6
Wada‐Isoe et al. (2004)	Cross‐sectional	Japan	37 (26/11)	69.1	NA	IL‐6 (CSF, ELISA)	AD: DSM‐III‐R, NINCDS‐ADRDA criteria and HIS≤4; VaD: DSM‐III‐R, ADDTC criteria and HIS≥7	5
Jia et al. (2005)	Cross‐sectional	China	77 (39/38)	65.4	51.9	IL‐1α, IL‐1β, IL‐2, IL‐6, TNF‐α (CSF, ELISA)	AD: NINCDS‐ADRDA criteria; VaD: NINDS‐AIREN criteria	6
Zuliani et al. (2007)	Cross‐sectional	Italy	140 (60/80)	78.8	41.4	IL‐6, TNF‐α, IL‐1β, IL‐10 (plasma, ELISA)	AD: NINCDS‐ADRDA criteria; VaD: NINDS‐AIREN criteria	7
Mancinella et al. (2009)	Cross‐sectional	Italy	99 (35/64)	84.0	25.3	CRP (serum, near infrared particle immunoassay)	AD: NINCDS‐ADRDA criteria; VaD: NINDS‐AIREN criteria	6
L. Li et al. (2010)	Cross‐sectional	Germany	44 (28/16)	80.6	38.6	CRP (plasma, ELISA)	AD: NINCDS‐ADRDA criteria; VaD: NINDS‐AIREN criteria	6
Uslu et al. (2012)	Cross‐sectional	Turkey	44 (28/16)	69.6	38.6	IL‐6, TNF‐α (serum, ELISA)	AD: NINCDS‐ADRDA criteria; VaD: NINDS‐AIREN criteria	6
Dukic et al. (2016)	Cross‐sectional	Croatia	137 (70/67)	76.0	39.4	CRP, IL‐6 (serum, Beckman Coulter 2700 biochemistry analyzer; electrochemiluminescence immunochemistry analyzer Cobas e411)	AD: NIA‐AA 2011 criteria; VaD: NINDS‐AIREN criteria	7
Vishnu et al. (2017)	Cross‐sectional	India	52 (41/11)	NA	NA	CRP, IL‐6 (plasma, ELISA)	AD: dubois criteria; VaD: DSM IV criteria	5
Wehr et al. (2019)	Cross‐sectional	Poland	251 (166/85)	72.9	32.7	CRP, IL‐6 (serum, ELISA)	AD: NINCDS‐ADRDA criteria; VaD: NINDS‐AIREN criteria	6
Chua et al. (2020)	Case‐cohort	Singapore	144 (113/31)	75.5	38.9	IL‐6, TNF‐α (serum, Luminex immunoassays)	AD: NINCDS‐ADRDA criteria; VaD: NINDS‐AIREN criteria	7
Lauriola et al. (2021)	Cross‐sectional	Italy	465 (267/198)	70.6	36.1	CRP (serum, NA)	AD: NINCDS‐ADRDA criteria; VaD: NINDS‐AIREN criteria	6
Khatri et al. (2024)	Cross‐sectional	India	66 (44/22)	66.9	59.1	CRP, IL‐6 (serum, ELISA)	AD: NINCDS‐ADRDA criteria; VaD: NINDS‐AIREN criteria	6

### 3.2 Study Characteristics

Of the 15 included studies, 14 employed a cross‐sectional design and 1 used a case‐cohort design. Nine studies were conducted in Western countries and six in Eastern countries. The total sample comprised 1728 participants, with individual study sizes ranging from 37 to 465 participants. Study quality, assessed using the NOS, classified three studies as high quality (scoring 7 points) and the remaining 12 as moderate quality (nine studies scoring 6 points and three scoring 5 points).

### 3.3 Il‐6

**FIGURE 2:**
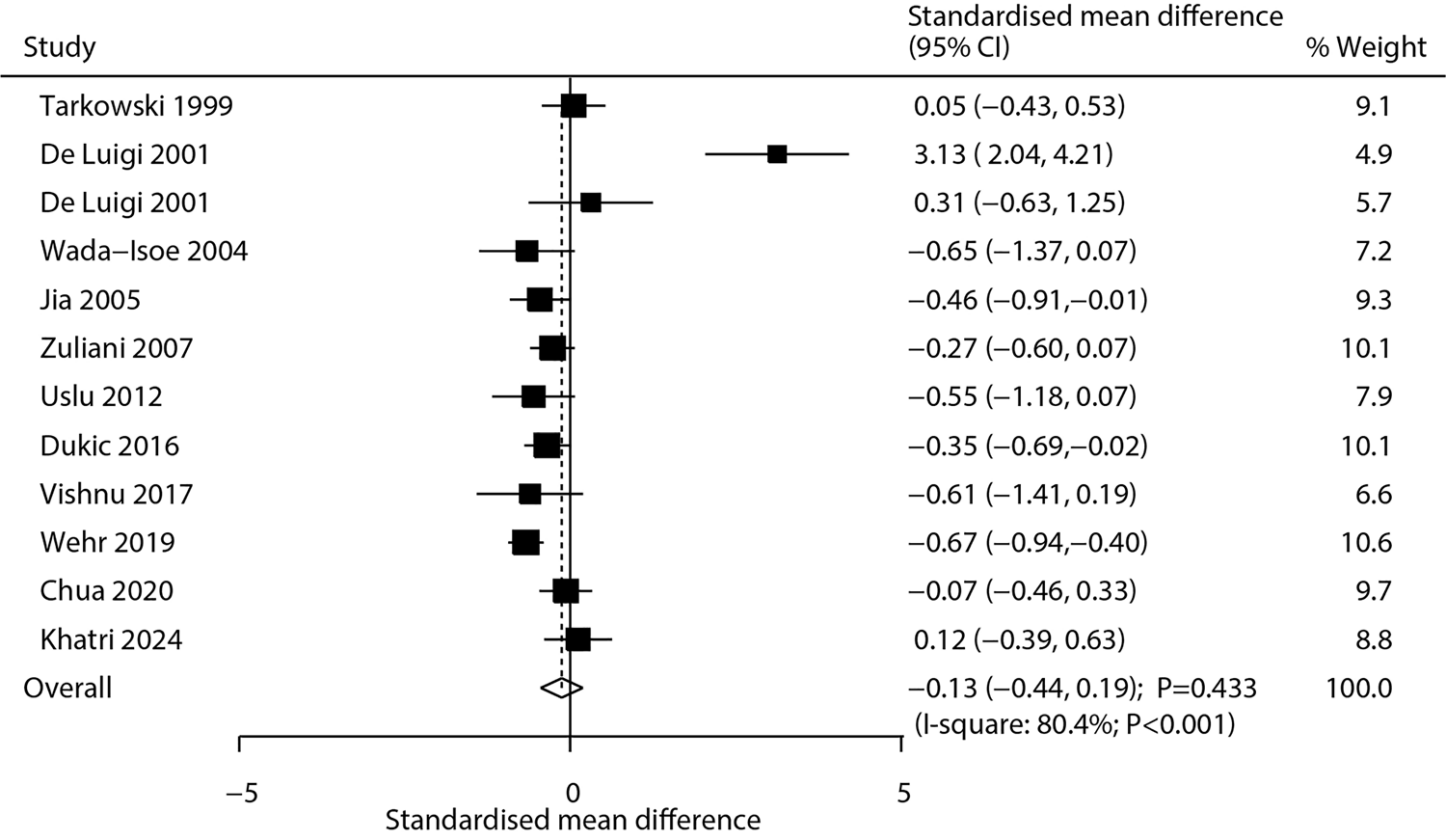
Pooled analysis of IL‐6 level differences between patients with AD and patients with VaD.

Eleven studies reported differences in IL‐6 levels between patients with AD and patients with VaD. The pooled analysis showed no statistically significant difference (SMD: −0.13; 95% CI: −0.44 to 0.19; *p* = 0.433; Figure 2), with substantial heterogeneity (*I* ^2^ = 80.4%; *p* < 0.001). Sensitivity analysis suggested IL‐6 levels in patients with AD might be lower than in patients with VaD (Supporting Information 2 [Figure S1]). Subgroup analysis indicated potentially lower IL‐6 levels in AD patients in studies from Eastern countries, with sample sizes ≥100, mean patient age ≥70 years, reported cerebrospinal fluid (CSF) IL‐6, or higher methodological quality (Table 2). Funnel plot inspection and Egger's (*p* = 0.061) and Begg's (*p* = 0.304) tests detected no significant publication bias (Supporting Information 3 [Figure S1]).

**TABLE 2:** Subgroup analyses for AD versus VaD on inflammatory biomarkers.

Biomarkers	Factors	Subgroup	SMD and 95%CI	*p* value	*I* ^2^ (%)	*Q* statistic	Interaction test
IL‐6	Country	Eastern	−0.29 (−0.55 to −0.44)	0.023	22.5	0.264	0.837
		Western	0.17 (−0.40 to 0.74)	0.563	89.9	< 0.001	
	Sample size	≥ 100	−0.36 (−0.62 to −0.11)	0.006	58.9	0.063	0.031
		< 100	0.08 (−0.49 to 0.65)	0.787	84.1	< 0.001	
	Mean age (years)	≥ 70	−0.36 (−0.62 to −0.11)	0.006	58.9	0.063	0.002
		< 70	−0.26 (−0.57 to 0.05)	0.103	38.0	0.168	
	Biofluid type	Serum	−0.27 (−0.55 to 0.01)	0.062	63.8	0.017	0.078
		Plasma	0.58 (−0.71 to 1.86)	0.379	91.9	< 0.001	
		CSF	−0.51 (−0.90 to −0.13)	0.009	0.0	0.656	
	Study quality	High	−0.25 (−0.45 to −0.04)	0.019	0.0	0.551	0.539
		Moderate	−0.03 (−0.52 to 0.45)	0.893	85.3	< 0.001	
TNF‐α	Country	Eastern	−0.64 (−1.30 to 0.01)	0.055	80.8	0.005	0.007
		Western	0.13 (−0.60 to 0.86)	0.726	87.1	< 0.001	
	Sample size	≥ 100	−0.21 (−0.47 to 0.05)	0.107	0.0	0.911	0.334
		< 100	−0.19 (−1.01 to 0.64)	0.660	90.0	< 0.001	
	Mean age (years)	≥ 70	−0.33 (−0.63 to −0.03)	0.033	34.5	0.217	< 0.001
		< 70	−0.54 (−1.37 to 0.28)	0.198	86.5	0.001	
	Biofluid type	Serum	−0.30 (−0.67 to 0.06)	0.102	51.0	0.106	< 0.001
		Plasma	0.70 (−1.19 to 2.59)	0.469	94.0	< 0.001	
		CSF	−1.23 (−1.72 to −0.74)	< 0.001	—	—	
	Study quality	High	−0.21 (−0.47 to 0.05)	0.107	0.0	0.911	0.334
		Moderate	−0.19 (−1.01 to 0.64)	0.660	90.0	< 0.001	
CRP	Country	Eastern	0.02 (−0.49 to 0.53)	0.946	—	—	0.389
		Western	0.89 (−0.15 to 1.93)	0.095	97.8	< 0.001	
	Sample size	≥ 100	−0.44 (−0.78 to −0.10)	0.011	80.4	0.006	< 0.001
		< 100	2.14 (−0.93 to 5.20)	0.172	98.4	< 0.001	
	Mean age (years)	≥ 70	0.89 (−0.15 to 1.93)	0.095	97.8	< 0.001	0.389
		< 70	0.02 (−0.49 to 0.53)	0.946	—	—	
	Biofluid type	Serum	0.81 (−0.20 to 1.82)	0.117	97.8	< 0.001	0.071
		Plasma	0.36 (−0.26 to 0.98)	0.258	—	—	
	Study quality	High	−0.42 (−0.76 to −0.08)	0.015	—	—	0.169
		Moderate	1.00 (−0.13 to 2.13)	0.083	97.8	< 0.001	
IL‐1β	Country	Eastern	—	—	—	—	—
		Western	0.48 (0.18 to 0.79)	0.002	0.0	0.425	
	Sample size	≥ 100	—	—	—	—	—
		< 100	0.48 (0.18 to 0.79)	0.002	0.0	0.425	
	Mean age (years)	≥ 70	0.78 (0.29 to 1.27)	0.002	0.0	0.670	0.188
		< 70	0.46 (−0.03 to 0.95)	0.063	—	—	
	Biofluid type	Serum	0.62 (0.28 to 0.96)	< 0.001	0.0	0.598	0.114
		Plasma	0.05 (−0.57 to 0.67)	0.872	0.0	0.560	
	Study quality	High	—	—	—	—	—
		Moderate	0.48 (0.18 to 0.79)	0.002	0.0	0.425	

### 3.4 TNF‐α

**FIGURE 3:**
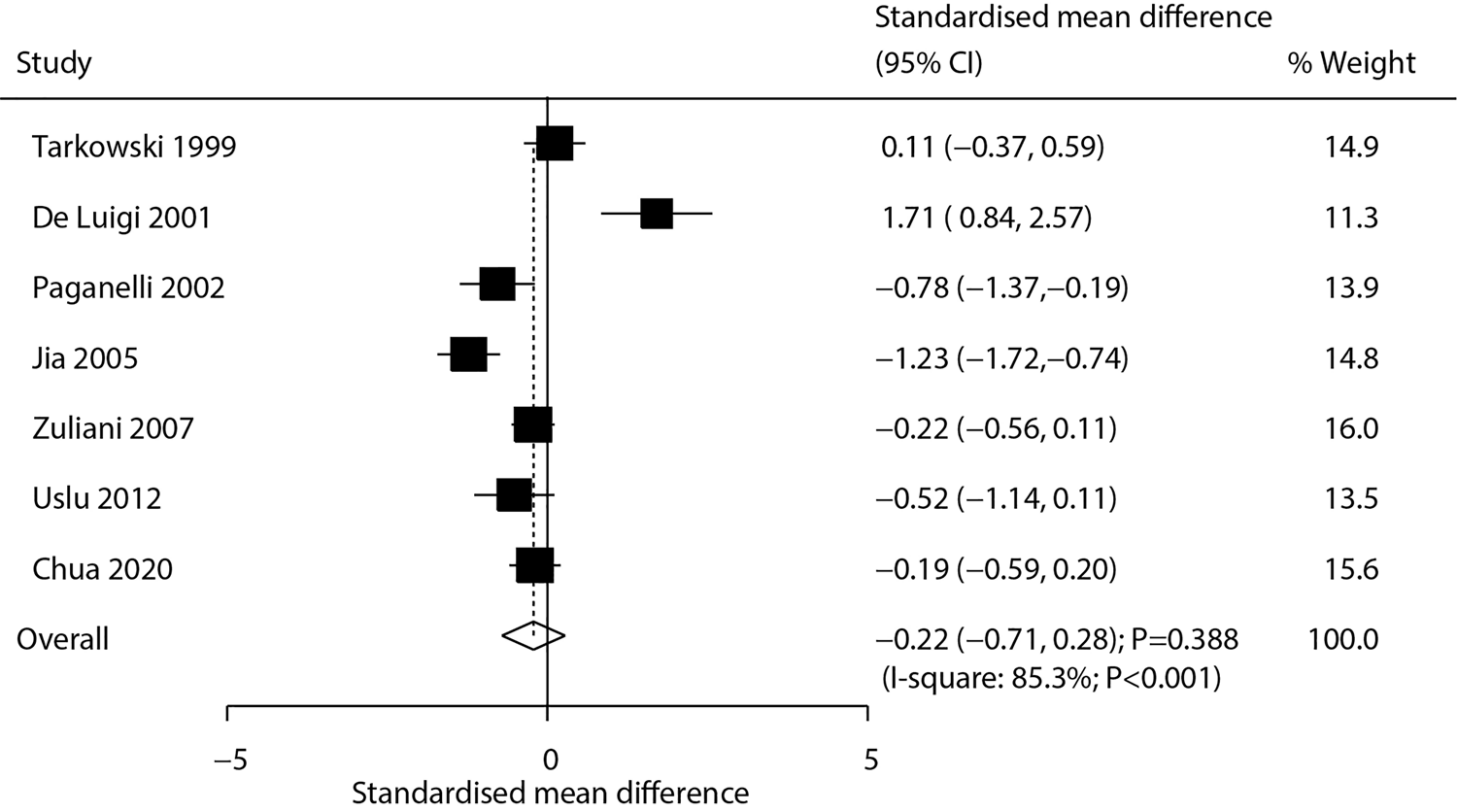
Pooled analysis of TNF‐α level differences between patients with AD and patients with VaD.

Seven studies reported TNF‐α level differences between AD and patients with VaD. The pooled analysis found no significant difference (SMD: −0.22; 95% CI: −0.71 to 0.28; *p* = 0.388; Figure 3), with considerable heterogeneity (*I* ^2^ = 85.3%; *p* < 0.001). Sensitivity analysis again suggested that TNF‐α levels in patients with AD might be lower than in patients with VaD (Supporting Information 2 [Figure S2]). Subgroup analysis revealed significantly lower TNF‐α levels in patients with AD when restricted to studies with a mean patient age ≥70 years, or reported CSF TNF‐α (Table 2). No significant publication bias was detected by funnel plot analysis, Egger's test (*p* = 0.561), or Begg's test (*p* = 0.764) (Supporting Information 3 [Figure S2]).

### 3.5 Crp

**FIGURE 4:**
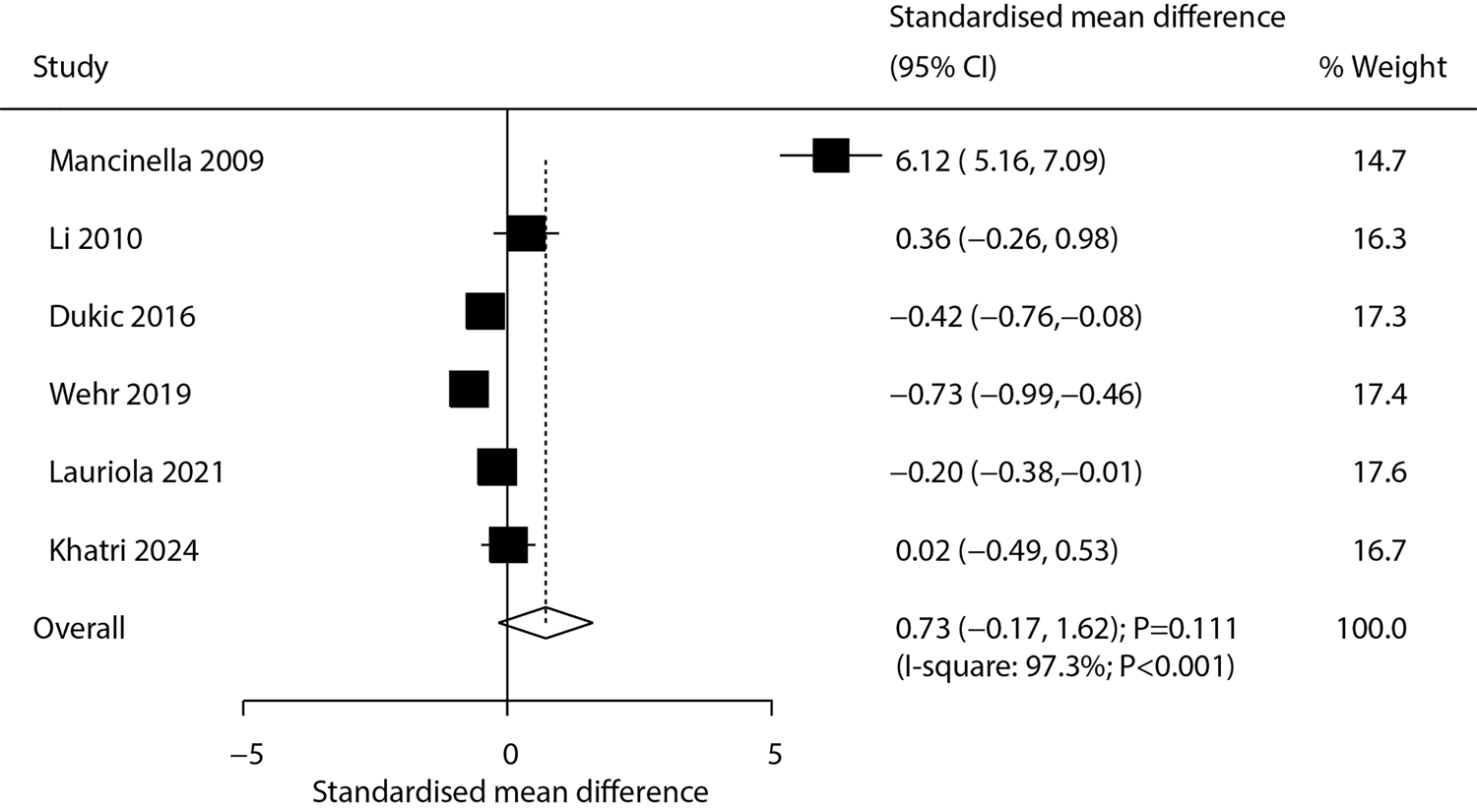
Pooled analysis of CRP level differences between patients with AD and patients with VaD.

Six studies reported CRP level differences between AD and patients with VaD. The pooled analysis showed no statistically significant difference (SMD: 0.73; 95% CI: −0.17 to 1.62; *p* = 0.111; Figure 4), with extremely high heterogeneity (*I* ^2^ = 97.3%; *p* < 0.001). Sensitivity analysis indicated no significant difference (Supporting Information 2 [Figure S3]). Subgroup analysis showed significantly lower CRP levels in patients with AD in studies with sample sizes ≥100 or higher‐quality methodologies (Table 2). Funnel plot evaluation and Egger's (*p* = 0.148) and Begg's (*p* = 0.060) tests indicated no significant publication bias (Supporting Information 3 [Figure S3]).

### 3.6 IL‐1β

**FIGURE 5:**
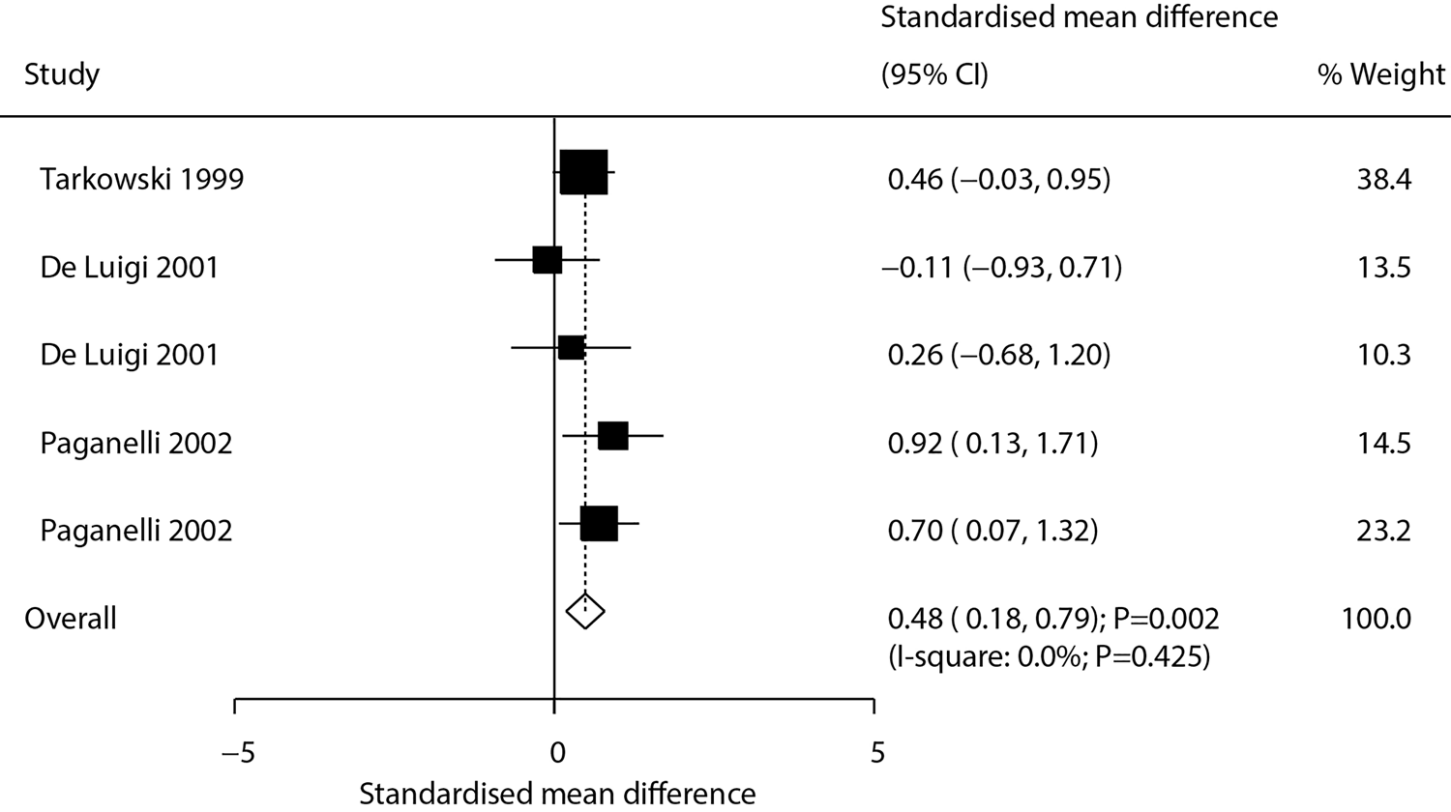
Pooled analysis of IL‐1β level differences between patients with AD and patients with VaD.

Three studies reported IL‐1β level differences between AD and patients with VaD. The pooled analysis revealed significantly higher IL‐1β levels in patients with AD (SMD: 0.48; 95% CI: 0.18 to 0.79; *p* = 0.002; Figure 5), with no observed heterogeneity (*I* ^2^ = 0.0%; *p* = 0.425). Sensitivity analysis confirmed the robustness of this result, showing it was not unduly influenced by any single study (Supporting Information 2 [Figure S4]). Subgroup analysis indicated no significant difference in IL‐1β levels when limited to studies with a mean patient age <70 years, or reported plasma IL‐1β (Table 2). Funnel plot assessment and Egger's (*p* = 0.711) and Begg's (*p* = 0.806) tests detected no significant publication bias (Supporting Information 3 [Figure S4]).

## 4 Discussion

This systematic review and meta‐analysis evaluated the diagnostic potential of inflammatory biomarkers in distinguishing VaD from AD. The findings indicated no significant overall differences in IL‐6, TNF‐α, and CRP levels between patients with AD and patients with VaD. However, subgroup analyses revealed context‐dependent variations influenced by methodological factors such as sample size, geographic region, patient age, and study quality. In contrast, IL‐1β levels were significantly higher in patients with AD compared to patients with VaD, with minimal heterogeneity, indicating consistent and robust pooled estimates.

For IL‐6, TNF‐α, and CRP, the absence of significant differences contrasts with earlier studies (Custodero et al. 2022). This inconsistency may stem from substantial heterogeneity observed across studies (IL‐6: *I* ^2^ = 80.4%; TNF‐α: *I* ^2^ = 85.3%; CRP: *I* ^2^ = 97.3%), likely attributable to several factors. The patterns of lower levels in AD for IL‐6 and TNF‐α emerged consistently under specific conditions: In studies conducted in Eastern populations, with larger sample sizes (≥100), involving older patients (≥70 years), or employing higher methodological quality. These factors likely reduce noise and increase the statistical power to detect disease‐specific pathophysiological differences. Importantly, the biomarker's biological compartment was a critical moderator. For IL‐6 and TNF‐α, the significant difference in favor of lower levels in AD was particularly pronounced in studies measuring these cytokines in CSF, compared to those using blood‐based matrices (plasma or serum). This strongly suggests that CSF levels more directly reflect the central neuroinflammatory processes characteristic of AD, whereas peripheral levels are confounded by systemic inflammation and vascular comorbidities more prevalent in VaD. Furthermore, for IL‐6, our additional analysis identified plasma (but not serum) as a biofluid source that could also reveal a significant AD versus VaD difference, highlighting the impact of pre‐analytical factors like platelet activation during clotting. The pattern for CRP was similarly influenced by study rigor (sample size, quality) but remained highly variable regardless of biofluid source, consistent with its role as a non‐specific acute‐phase reactant heavily influenced by systemic vascular burden. In contrast, IL‐1β levels were significantly and consistently higher in AD across studies, with minimal heterogeneity. Its diagnostic utility, however, was attenuated in studies focusing on younger patients (<70 years) or those measuring it in plasma, suggesting that the AD‐specific IL‐1β signal is most robust in older populations and may be diluted or less specific in peripheral circulation compared to its presumed central nervous system origin. Collectively, these subgroup findings underscore that the inflammatory profiles of AD and VaD are not monolithic. They are modulated by a combination of disease stage (reflected by age), genetic/environmental background (region), methodological precision (sample size, quality), and, most critically, the sampled biological compartment (CSF vs. blood, and plasma vs. serum) (Hu et al. 2025; Cheng et al. 2024; Puranik and Song 2025). The stronger discriminative power observed in CSF for IL‐6 and TNF‐α underscores the premise that biomarkers proximal to the brain pathology offer greater specificity for differential diagnosis.

IL‐1β showed a significant elevation in patients with AD compared to patients with VaD, with near‐zero heterogeneity, indicating exceptionally consistent and reproducible findings. This biomarker pattern is mechanistically linked to AD‐specific neuroinflammatory processes: Aβ plaque accumulation activates microglia via pattern recognition receptors such as NLRP3 inflammasomes, promoting sustained IL‐1β release (Z. Li and Gong 2025). This cytokine intensifies neuroinflammation through a feedback loop, promoting neuronal tau hyperphosphorylation, impairing synaptic plasticity, and accelerating neurodegeneration—a cascade less characteristic of VaD, where cerebrovascular damage primarily drives TNF‐α and CRP‐mediated endothelial inflammation rather than parenchymal IL‐1β activation (Ahmed 2025). However, subgroup analysis revealed that IL‐1β’s diagnostic utility was age‐dependent, losing significance in studies with a mean patient age below 70 years. This may reflect age‐related neuroinflammatory dynamics, as younger AD patients may represent earlier disease stages with incomplete amyloid‐induced microglial activation. In addition, aging may amplify baseline inflammation (“inflamm‐aging”), potentially masking disease‐specific cytokine patterns (Sun et al. 2022). Alternatively, younger patients with VaD might present with more heterogeneous vascular etiologies, differing from the small vessel disease predominating in older populations, thus influencing their inflammatory profiles.

The findings of this meta‐analysis, particularly the context‐dependent signals from classical cytokines and the potential of IL‐1β, highlight both the promise and the limitations of peripheral inflammatory markers. To advance diagnostic precision, future strategies should aim for multi‐modal biomarker integration. As introduced earlier, EVs represent a transformative platform that may overcome the specificity limitations of systemic cytokines. For instance, a 2023 meta‐analysis confirmed that neuron‐derived EVs carrying phosphorylated tau significantly differentiate AD from controls with higher accuracy than plasma tau (Zheng et al. 2025). Building on this, profiling inflammatory cargo within glial‐derived EVs (e.g., IL‐1β, specific miRNAs) in conjunction with the systemic markers analyzed here could more precisely isolate neuroinflammation central to AD from the vascular inflammation prevalent in VaD. Combining such EV‐based signals with clinical neuroimaging and the most robust circulating biomarkers could form the basis for a pathophysiology‐driven diagnostic algorithm, directly addressing the clinical challenges outlined at the outset of this review.

This review has several limitations. First, as pre‐specified, the primary analyses for IL‐6 did not show significant overall differences, guiding our main conclusion. The findings for TNF‐α, CRP, and IL‐1β are from secondary analyses, and all subgroup analyses are exploratory; thus, these results should be interpreted as hypothesis‐generating. Second, most included studies (14 out of 15) were cross‐sectional, limiting the ability to establish causal relationships between inflammatory biomarkers and dementia progression. Third, methodological variability—including differences in cohort characteristics (age, comorbidities), biomarker measurement techniques, and diagnostic criteria—introduced significant heterogeneity, affecting the generalizability of pooled results. Fourth, methodological variability in assay platforms, sample handling, and biofluid matrices, as detailed in Table S1, substantially contributes to the high statistical heterogeneity observed and limits the quantitative comparability of absolute biomarker levels across studies. Fifth, the small number of studies examining specific biomarkers, particularly IL‐1β (only three studies), reduced statistical power and increased susceptibility to outlier effects. Finally, while funnel plots and Egger's regression tests suggested no significant publication bias, the possibility of undetected bias remains, particularly given the likelihood of unpublished negative findings in emerging biomarker research.

## 5 Conclusions

This systematic review provides a preliminary synthesis of the evidence regarding inflammatory biomarkers in differentiating AD from VaD. The primary meta‐analyses did not reveal significant overall differences for IL‐6, TNF‐α, or CRP. Exploratory subgroup analyses hinted at context‐dependent variations, but these findings are constrained by extreme heterogeneity, potential confounding in the primary studies, and the inherent limitations of cross‐sectional data. Notably, secondary analysis of IL‐1β, based on only three studies, indicated higher levels in AD, positioning it as a biomarker candidate worthy of targeted investigation. However, this conclusion is preliminary and cannot support current clinical diagnostic use.

Critical limitations, including the small number of studies for key biomarkers, reliance on heterogeneous cross‐sectional designs, and variability in diagnostic and laboratory methods, preclude definitive claims about diagnostic utility. The current evidence base is best viewed as generating hypotheses for future research, not as validating clinical tools. To translate these exploratory insights into potential clinical relevance, future work must prioritize large, prospective cohort studies with pathologically confirmed diagnoses, standardized pre‐analytical and analytical protocols, and rigorous control for vascular and systemic confounders. The ultimate goal is to determine if inflammatory biomarkers, potentially in combination with other modalities, can reliably augment clinical diagnosis.

## Author Contributions

Study concept and design: Jiao Sun. Acquisition of data: Yingchun Ling, Lingmin Hu, and Mingyong Zhao. Analysis and interpretation of data: Yingchun Ling, Jiao Sun, Jie Chen, and Yating Zheng. Drafting of the manuscript: Yingchun Ling. Critical revision of the manuscript: Jiao Sun, Lingmin Hu, Mingyong Zhao, Jie Chen, Yating Zheng.

## Funding

The author(s) declare that financial support was received for the research and/or publication of this article. This study supported by “To explore the mechanism of the NLRP3caspase‐1GSDMD pathway mediated by miR‐146a‐5p to regulate the progression of Alzheimer's disease based on the inflammatory microenvironment (No: 2023A14029)” and “Study on the mechanism of regulating microglia mitochondria autophagy of Ginseng Yangrong Decoction to improve cognitive ability of Alzheimer's disease (No: 2024ZL1138)”.

## Conflicts of Interest

The authors declare no conflicts of interest.

## Supporting information




**Supplementary File 1**: brb371341‐sup‐0001‐SuppMat.docx

**Supplementary File 2**: brb371341‐sup‐0002‐SuppMat.docx

**Supplementary File 3**: brb371341‐sup‐0003‐SuppMat.docx

## Data Availability

The original contributions presented in the study are included in the article/supplementary material, further inquiries can be directed to the corresponding authors.
