# Supplementary material for: Diagnostic Value of Inflammatory Biomarkers in Differentiating Vascular Dementia From Alzheimer's Disease: A Systematic Review and Meta‐Analysis
Source: Brain Behav. 2026 Apr 16;16(4):e71341. doi: 10.1002/brb3.71341 (PMC13087516; doi:10.1002/brb3.71341)
Supplement: Supplementary file 1 — brb371341‐sup‐0001‐SuppMat.docx [file BRB3-16-e71341-s001.docx]

**Search strategies:**

**PubMed:**

#1 "Alzheimer Disease"[Mesh]

#2 (Alzheimer*[tiab]) OR (AD[tiab])

#3 #1 OR #2

#4 "Vascular Dementia"[Mesh]

#5 "Vascular Cognitive Impairment"[Mesh]

#6 (Vascular Cognitive Impairment*[tiab]) OR (VCID[tiab])

#7 (Vascular Cognitive Disorder*[tiab])

#8 (Vascular Dementia[tiab]) OR (VaD[tiab])

#9 (Multi-Infarct Dementia[tiab]) OR (Subcortical Ischemic Vascular Dementia[tiab]) OR (SIVD[tiab])

#10 (Post-Stroke Dementia[tiab]) OR (Poststroke Dementia[tiab])

#11 #4 OR #5 OR #6 OR #7 OR #8 OR #9 OR #10

#12 "Inflammation"[Mesh] OR "Neuroinflammation"[Mesh]

#13 "C-Reactive Protein"[Mesh] OR CRP[tiab] OR "c reactive protein"[tiab]

#14 "Interleukin-1"[Mesh] OR "IL-1"[tiab] OR "Interleukin 1"[tiab] OR IL1beta[tiab] OR IL-1β[tiab] OR IL1β[tiab]

#15 "Interleukin-6"[Mesh] OR "IL-6"[tiab] OR "Interleukin 6"[tiab]

#16 "Tumor Necrosis Factor-alpha"[Mesh] OR TNF-alpha[tiab] OR TNFα[tiab] OR "TNF alpha"[tiab]

#17 "Interleukin-8"[Mesh] OR "IL-8"[tiab] OR CXCL8[tiab]

#18 "Interferon-gamma"[Mesh] OR IFN-gamma[tiab] OR IFNγ[tiab] OR "IFN gamma"[tiab]

#19 "Interleukin-17"[Mesh] OR "IL-17"[tiab] OR "Interleukin 17"[tiab]

#20 "Interleukin-12"[Mesh] OR "IL-12"[tiab] OR "Interleukin 12"[tiab]

#21 "Interleukin-10"[Mesh] OR "IL-10"[tiab] OR "Interleukin 10"[tiab]

#22 "Interleukin-18"[Mesh] OR "IL-18"[tiab]

#23 "Interleukin-4"[Mesh] OR "IL-4"[tiab]

#24 "Interleukin-13"[Mesh] OR "IL-13"[tiab]

#25 "Chemokines"[Mesh] OR Chemokine*[tiab] OR CCL2[tiab] OR MCP-1[tiab] OR CX3CL1[tiab] OR Fractalkine[tiab]

#26 "Biomarkers"[Mesh] OR biomarker*[tiab] OR "biological marker*"[tiab]

#27 Cytokine*[tiab] OR Inflammatory Marker*[tiab]

#28 #12 OR #13 OR #14 OR #15 OR #16 OR #17 OR #18 OR #19 OR #20 OR #21 OR #22 OR #23 OR #24 OR #25 OR #26 OR #27

#29 #3 AND #11 AND #28

#30 Filters applied to #29: Humans

**Embase:**

1. exp Alzheimer disease/

2. (Alzheimer* or AD).ti,ab.

3. 1 or 2

4. exp vascular dementia/

5. exp cerebrovascular disease/

6. (vascular cognitive impairment* or VCID).ti,ab.

7. (vascular cognitive disorder*).ti,ab.

8. (vascular dementia or VaD).ti,ab.

9. (multi infarct dementia or subcortical ischemic vascular dementia or SIVD).ti,ab.

10. (post stroke dementia or poststroke dementia).ti,ab.

11. 4 or 5 or 6 or 7 or 8 or 9 or 10

12. exp inflammation/ or exp neuroinflammation/

13. exp C reactive protein/ or (CRP or c reactive protein).ti,ab.

14. exp interleukin 1/ or (IL-1 or Interleukin 1 or IL1beta or IL-1β or IL1β).ti,ab.

15. exp interleukin 6/ or (IL-6 or Interleukin 6).ti,ab.

16. exp tumor necrosis factor alpha/ or (TNF-alpha or TNFα or TNF alpha).ti,ab.

17. exp interleukin 8/ or (IL-8 or CXCL8).ti,ab.

18. exp interferon gamma/ or (IFN-gamma or IFNγ or IFN gamma).ti,ab.

19. exp interleukin 17/ or (IL-17 or Interleukin 17).ti,ab.

20. exp interleukin 12/ or (IL-12 or Interleukin 12).ti,ab.

21. exp interleukin 10/ or (IL-10 or Interleukin 10).ti,ab.

22. exp interleukin 18/ or (IL-18).ti,ab.

23. exp chemokine/ or (chemokine* or CCL2 or MCP-1 or CX3CL1 or Fractalkine).ti,ab.

24. exp biological marker/ or (biomarker* or biological marker*).ti,ab.

25. (cytokine* or inflammatory marker*).ti,ab.

26. 12 or 13 or 14 or 15 or 16 or 17 or 18 or 19 or 20 or 21 or 22 or 23 or 24 or 25

27. 3 and 11 and 26

28. limit 27 to human.

**Web of Science:**

#1 TS=(Alzheimer* OR AD)

#2 TS=("Vascular Dementia" OR VaD OR "Vascular Cognitive Impairment" OR VCID OR "Vascular Cognitive Disorder*" OR "Multi-Infarct Dementia" OR "Subcortical Ischemic Vascular Dementia" OR "Post-Stroke Dementia")

#3 TS=("inflammation" OR "neuroinflammation" OR "C-reactive protein" OR CRP OR "Interleukin-1" OR "IL-1" OR IL1beta OR "IL-1β" OR "Interleukin-6" OR "IL-6" OR "Tumor Necrosis Factor-alpha" OR TNF-alpha OR TNFα OR "Interleukin-8" OR "IL-8" OR CXCL8 OR "Interferon-gamma" OR IFN-gamma OR IFNγ OR "Interleukin-17" OR "IL-17" OR "Interleukin-12" OR "IL-12" OR "Interleukin-10" OR "IL-10" OR chemokine* OR CCL2 OR MCP-1 OR biomarker* OR cytokine*)

#4 #1 AND #2 AND #3

#5 Refined by: [DOCUMENT TYPES: (ARTICLE OR REVIEW)]

**Cochrane Library:**

#1 MeSH descriptor: [Alzheimer Disease] explode all trees

#2 (Alzheimer* or AD):ti,ab,kw

#3 #1 or #2

#4 MeSH descriptor: [Vascular Dementia] explode all trees

#5 MeSH descriptor: [Cognitive Dysfunction] this term only with qualifier(s): [etiology - ET, cerebrovascular disorders - CO]

#6 (vascular dementia or VaD):ti,ab,kw

#7 (vascular cognitive impairment or VCID):ti,ab,kw

#8 (vascular cognitive disorder*):ti,ab,kw

#9 (multi infarct dementia or subcortical ischemic vascular dementia):ti,ab,kw

#10 #4 or #5 or #6 or #7 or #8 or #9

#11 MeSH descriptor: [Inflammation] explode all trees

#12 MeSH descriptor: [C-Reactive Protein] explode all trees

#13 (CRP or "c reactive protein"):ti,ab,kw

#14 MeSH descriptor: [Interleukin-1] explode all trees

#15 (IL-1 or Interleukin-1 or IL1beta or "IL-1β"):ti,ab,kw

#16 MeSH descriptor: [Interleukin-6] explode all trees

#17 (IL-6 or Interleukin-6):ti,ab,kw

#18 MeSH descriptor: [Tumor Necrosis Factor-alpha] explode all trees

#19 (TNF-alpha or TNFα):ti,ab,kw

#20 (Interleukin-8 or IL-8 or CXCL8):ti,ab,kw

#21 (Interferon-gamma or IFN-gamma or IFNγ):ti,ab,kw

#22 (Interleukin-17 or IL-17):ti,ab,kw

#23 (Interleukin-12 or IL-12):ti,ab,kw

#24 (Interleukin-10 or IL-10):ti,ab,kw

#25 (chemokine* or CCL2 or MCP-1):ti,ab,kw

#26 (biomarker* or cytokine* or inflammatory marker*):ti,ab,kw

#27 #11 or #12 or #13 or #14 or #15 or #16 or #17 or #18 or #19 or #20 or #21 or #22 or #23 or #24 or #25 or #26

#28 #3 and #10 and #27
