# Supplementary material for: Diagnostic Value of Inflammatory Biomarkers in Differentiating Vascular Dementia From Alzheimer's Disease: A Systematic Review and Meta‐Analysis
Source: Brain Behav. 2026 Apr 16;16(4):e71341. doi: 10.1002/brb3.71341 (PMC13087516; doi:10.1002/brb3.71341)
Supplement: Supplementary file 2 — brb371341‐sup‐0002‐SuppMat.docx [file BRB3-16-e71341-s002.docx]

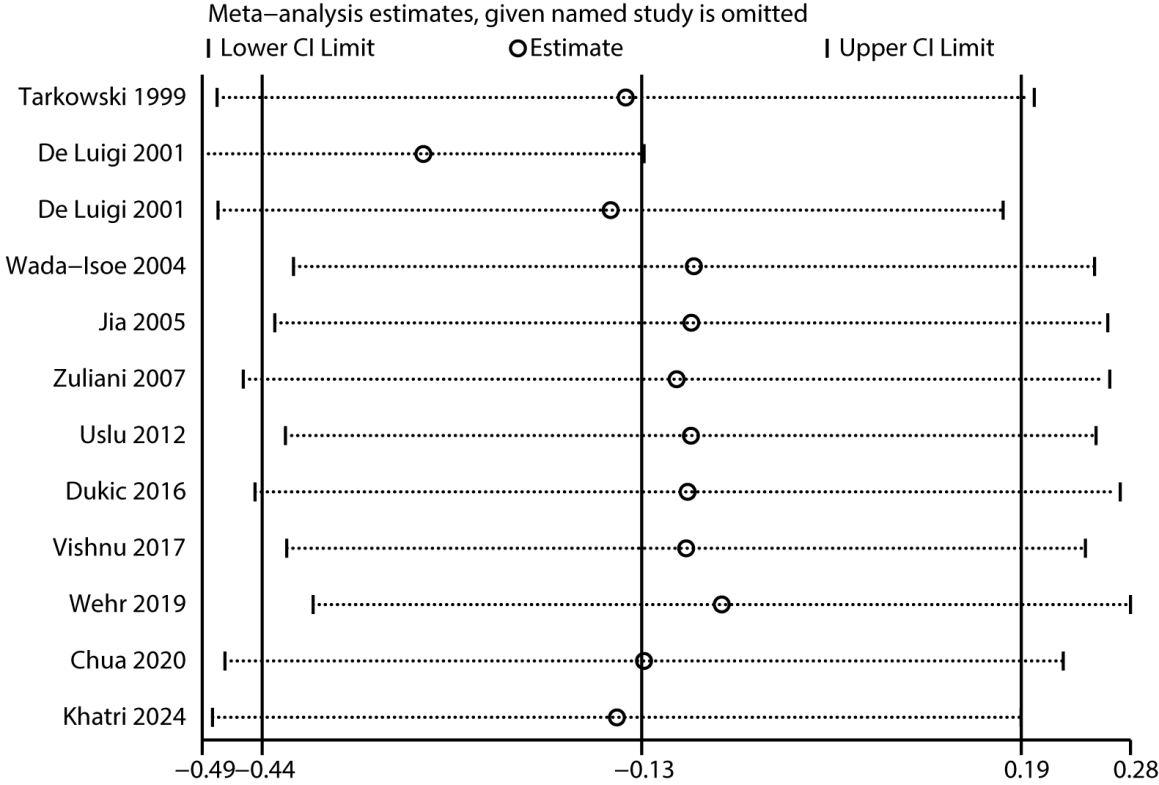


Figure S1. Sensitivity analysis for AD versus VaD on IL-6 level


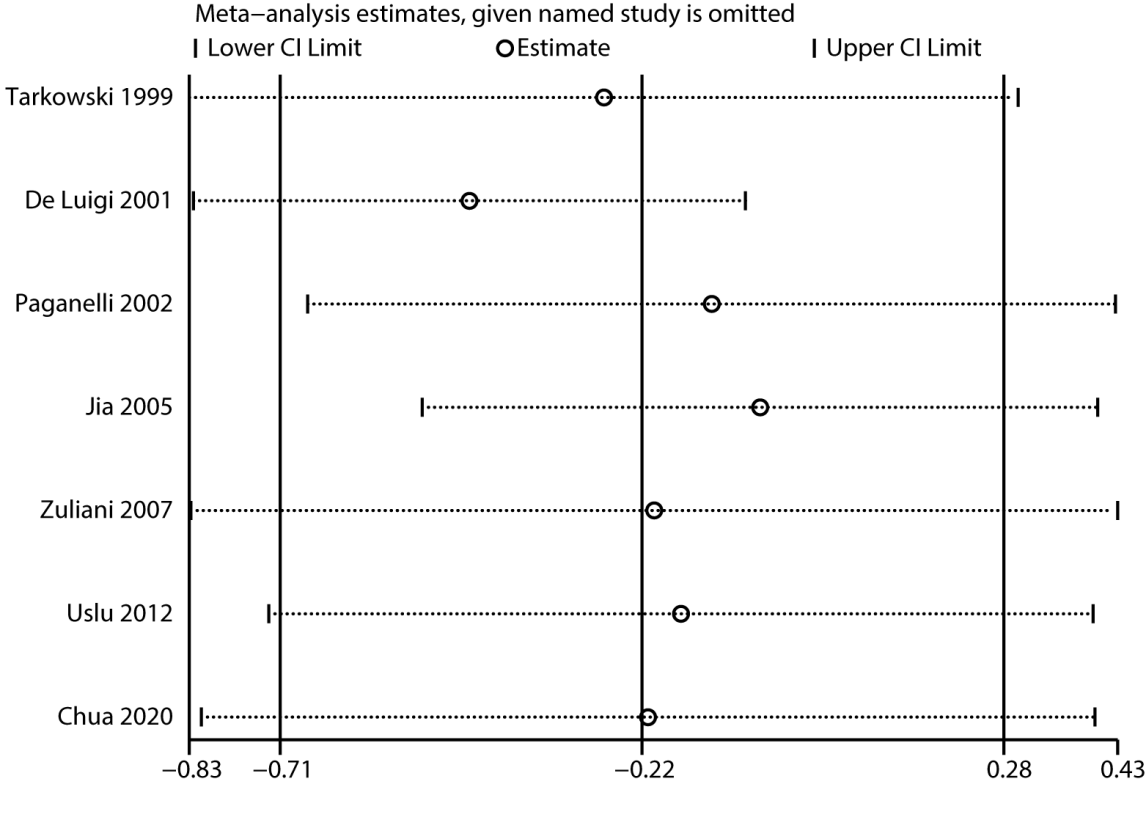


Figure S2. Sensitivity analysis for AD versus VaD on TNF-α


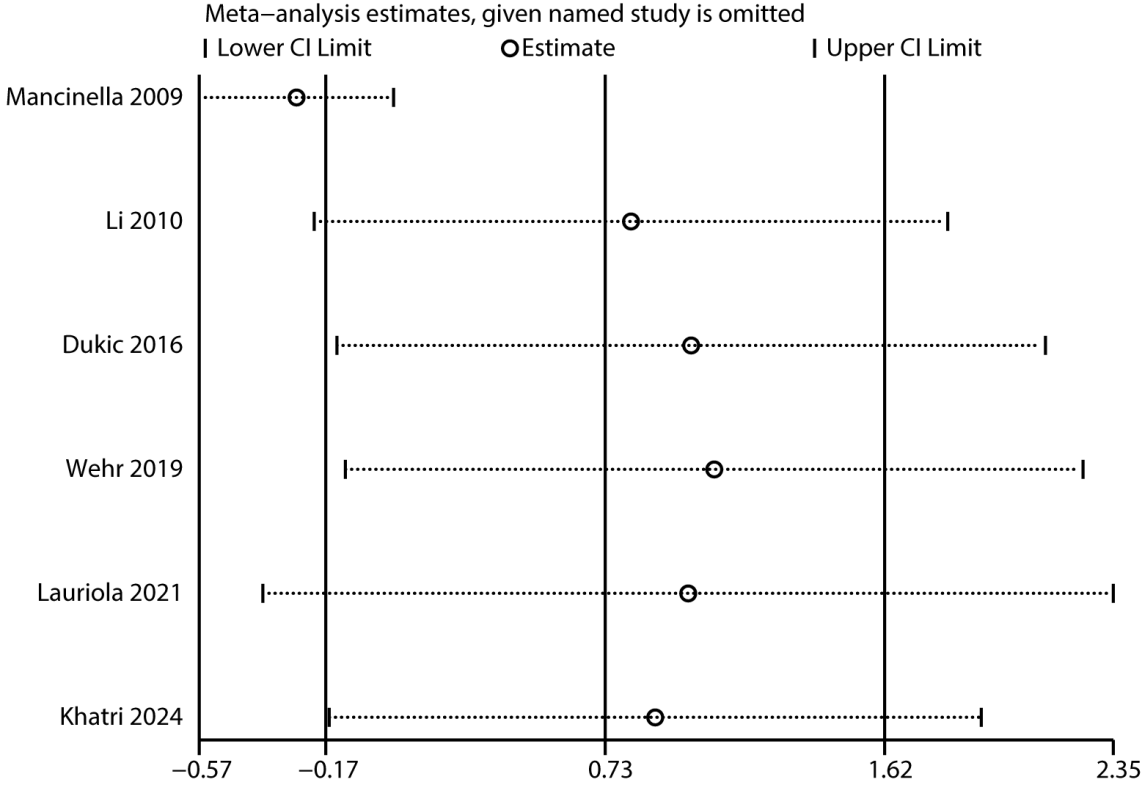


Figure S3. Sensitivity analysis for AD versus VaD on CRP


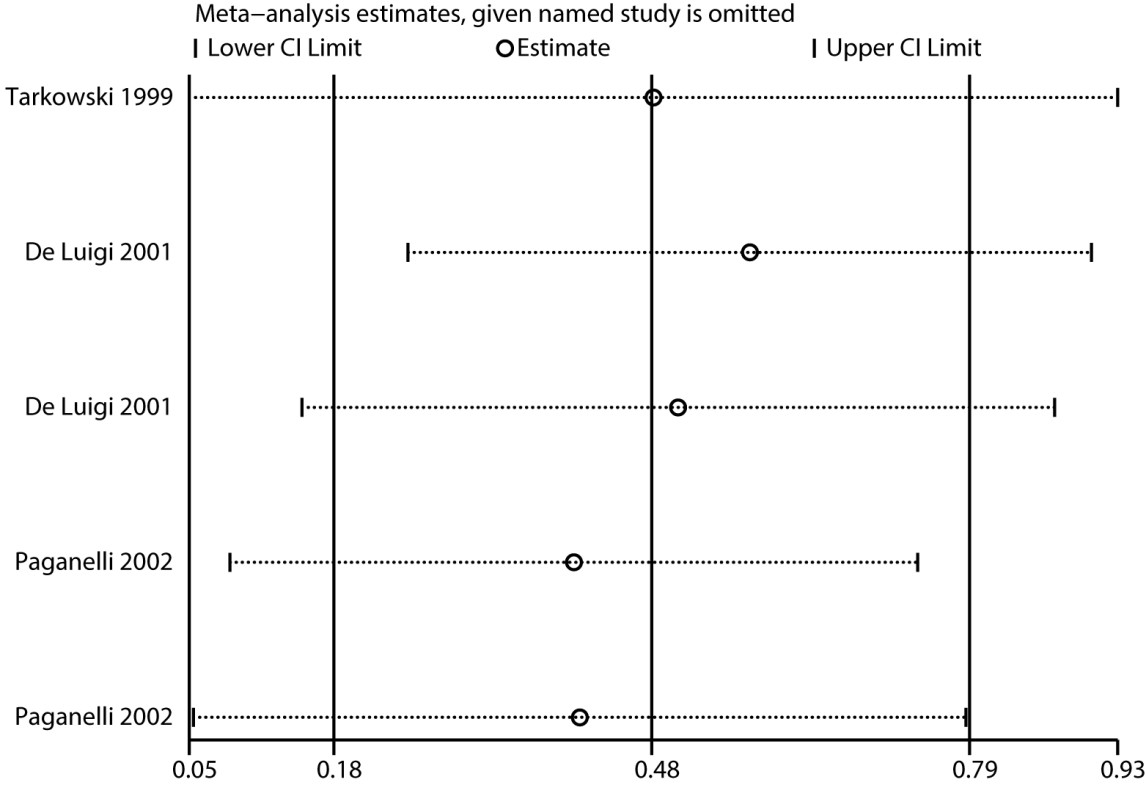


Figure S4. Sensitivity analysis for AD versus VaD on IL-1β
